# Real-time X-ray imaging of mouse cerebral microvessels *in vivo* using a pixel temporal averaging method

**DOI:** 10.1107/S1600577521012522

**Published:** 2022-01-01

**Authors:** Fucheng Yu, Feixiang Wang, Ke Li, Guohao Du, Biao Deng, Honglan Xie, Guoyuan Yang, Tiqiao Xiao

**Affiliations:** a Shanghai Institute of Applied Physics, Chinese Academy of Sciences, Shanghai 201204, People’s Republic of China; b Shanghai Synchrotron Radiation Facility/Zhang Jiang Lab, Shanghai Advanced Research Institute, Chinese Academy of Sciences, Shanghai 201800, People’s Republic of China; c University of Chinese Academy of Sciences, Beijing, People’s Republic of China; d Med-X Research Institute and School of Biomedical Engineering, Shanghai Jiao Tong University, Shanghai, People’s Republic of China

**Keywords:** synchrotron radiation, cerebral microvessels, X-ray angiography, motion artifacts

## Abstract

Rodents are used extensively as animal models for the preclinical investigation of microvascular-related diseases. However, motion artifacts in currently available imaging methods preclude real-time observation of microvessels *in vivo*. In this paper, a pixel temporal averaging (PTA) method that enables real-time imaging of microvessels in the mouse brain *in vivo* is described. Experiments using live mice demonstrated that PTA efficiently eliminated motion artifacts and random noise, resulting in significant improvements in contrast-to-noise ratio. The time needed for image reconstruction using PTA with a normal computer was 250 ms, highlighting the capability of the PTA method for real-time angiography. In addition, experiments with less than one-quarter of photon flux in conventional angiography verified that motion artifacts and random noise were suppressed and microvessels were successfully identified using PTA, whereas conventional temporal subtraction and averaging methods were ineffective. Experiments performed with an X-ray tube verified that the PTA method could also be successfully applied to microvessel imaging of the mouse brain using a laboratory X-ray source. In conclusion, the proposed PTA method may facilitate the real-time investigation of cerebral microvascular-related diseases using small animal models.

## Abstract 2

A pixel temporal averaging method is developed to eliminate motion artifacts and random noises aiming at real-time and *in vivo* radiology of mouse cerebral microvessels.

## 1. Introduction

Extant evidence suggests that cerebral microvascular dysfunction is associated with various diseases, such as cerebral ischemia, hemorrhagic stroke, and hypertension (Hughes & Bund, 2002 ▸; Wang *et al.*, 2017 ▸, 2019 ▸; Balbi *et al.*, 2015 ▸). Promoting angiogenesis is key for the repair and reconstruction of vessel function after ischemic stroke (Lu *et al.*, 2012 ▸). Rodent models are widely used for the preclinical investigation of microvascular-related cerebral diseases (Kidoguchi *et al.*, 2006 ▸; Kelly *et al.*, 2007 ▸; Chen *et al.*, 2014 ▸; Zhou *et al.*, 2019 ▸; Clark & Badea, 2021 ▸; Zhang *et al.*, 2019 ▸). In this regard, *in vivo* X-ray imaging of mouse brain microvessels constitutes a crucial aspect of relevant pathological studies.

Synchrotron radiation angiography is a commonly employed method for *in vivo* imaging of mouse brain microvasculature owing to its high flux density, which is beneficial for X-ray imaging with high temporal and spatial resolution (Kidoguchi *et al.*, 2006 ▸; Lin *et al.*, 2015 ▸; Liu *et al.*, 2010 ▸; Zhou *et al.*, 2019 ▸; Badea *et al.*, 2008 ▸). However, dense or thick background tissues strongly absorb X-rays, and their superimposition on blood vessels may interfere with the observation of blood vessels. Temporal subtraction techniques (Elleaume *et al.*, 2002 ▸; Suhonen *et al.*, 2008 ▸) are typically employed to address these issues. Temporal subtraction refers to obtaining a tissue-only image without injecting contrast agent as a mask map, then subtracting the mask image by the contrast image without contrast agent injection in order to obtain an undisturbed blood vessel image. However, the standard temporal subtraction method, which is commonly used to remove the interference of complex background signals resulting from the strong absorption of bones and soft tissues in the brain, inevitably suffers from motion artifacts and random noise when used in live animals. This causes deterioration of image contrast and makes it challenging to distinguish microvessels (Wang *et al.*, 2017 ▸). Motion artifacts are predominantly caused by the mismatch between the mask image and contrast image. To eliminate motion artifacts, the mask image and contrast image can be registered prior to subtraction. However, the registration method is unable to eliminate random noise (Maintz & Viergever, 1998 ▸; Meijering *et al.*, 1999 ▸), which precludes the explicit identification of microvessels. Generally, there are two main sources of random noise. Short circulation time prompts a high frame rate of X-ray cameras to improve the restored accuracy of blood flow velocity (Lin *et al.*, 2013 ▸, 2015 ▸; Hong *et al.*, 2012 ▸). As a result, short exposure time at a high frame rate introduces greater random noise. Further, during *in vivo* X-ray imaging in live animals, a beam filter is typically required to reduce the photon flux to ensure a low radiation dose. Fewer induced photons leads to an increase in random noise, which results in a lower contrast-to-noise ratio (CNR). To obtain clear blood vessel images, image denoising methods have been proposed, such as methods based on anisotropic or sparse decomposition (Perona & Malik, 1990 ▸; Candes *et al.*, 2006 ▸). However, the complex and time-consuming data processing associated with these methods has precluded their implementation for real-time observation of microvessels. In addition, the edge structure of vessel images can be partly blurred due to the denoising process. Noise reduction methods such as the application of a spatial mean filter for a selected subset of two-dimensional images have been employed, but this may lead to deterioration of spatial resolution (Tan & Jiang, 2019 ▸).

Move contrast X-ray imaging was recently proposed for the sensitive imaging of intact mouse microvessels *in vivo*. This method enables simultaneous elimination of motion artifacts and noise by tracking the contrast agent as it passes through the vessel lumen in the frequency domain. This approach has contributed to significant improvements in the sensitivity of imaging microvessels (Wang *et al.*, 2020 ▸). However, the time needed for image reconstruction is relatively long, which prevents the use of this method for real-time observation of microvessels *in vivo*. A real-time digital subtraction angiography method (Yamamoto *et al.*, 2009 ▸) that continuously changes the mask image is applied to cardioangiography to reduce motion artifacts, but this method cannot simultaneously reduce random noise. In addition, the conventional averaging method is frequently used in low-dose X-ray fluoro­scopy imaging to remove quantum noise (Wilson *et al.*, 1999 ▸). However, this method is hampered by motion blur during the *in vivo* imaging of microvessels. Stereotaxic frames or simple head restraints are typically employed to reduce motion artifacts. Nevertheless, the removal of motion blur of cerebral microvessels resulting from convulsion, heart beating, or breathing remains challenging.

In this study, we developed a pixel temporal averaging (PTA) method to simultaneously eliminate random noise and motion artifacts while performing real-time X-ray imaging of mouse cerebral microvessels *in vivo* by combining the traditional temporal averaging method with motion frequency analysis. First, we introduce the principles underlying this novel method. We then describe the validation of our proposed method using experiments in live mice. By suppressing the motion of background tissues and random noise, the image quality of microvessels was significantly improved, and distinct real-time visualization of microvasculature was achieved. Further, we verified the robustness of PTA in low-dose imaging of microvessels by reducing the photon flux incident in animal models.

## 2. Methods

### 2.1. Principle

We considered pixel intensity, which can be defined as a time-varying function in principle and expressed as

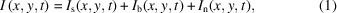

where *I*_s_(*x*, *y*, *t*) denotes the contribution of the signal of interest, *I*_b_(*x*, *y*, *t*) refers to the tissue background, *I*_n_(*x*, *y*, *t*) refers to the noise, and (*x*, *y*) denotes the coordinates of the pixel. Fluctuations in *I*_b_(*x*, *y*, *t*) and *I*_n_(*x*, *y*, *t*) lead to deterioration of the signal *I*_s_(*x*, *y*, *t*). Averaging across the entire time period will suppress these fluctuations but will simultaneously reduce signal contrast. The width of the time window used for averaging is critical for achieving an optimal CNR in our proposed method. Defining the window width as Δ*T* and number of windows as *i*,

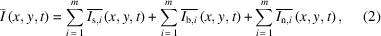

where 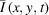 denotes the average of *I*(*x*, *y*, *t*) within the time window of Δ*T*, *m* is the total time period investigated, 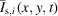 denotes the averaged value of *I*_s_(*x*, *y*, *t*) in the *i*th window, and similarly for 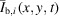 and 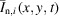 . Two factors affect the reconstructed image quality: the fidelity of the signal after averaging and the deviation of background and noise. Standard deviation (SD) is employed to evaluate the effect of background or noise suppression, resulting in

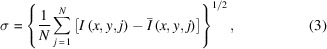

where *N* is the number of frames investigated, and *I*(*x*, *y*, *j*) and 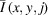 are the intensity at the *j*th frame and averaged intensity, respectively. In principle, the SD of *I*_b_(*x*, *y*) and *I*_n_(*x*, *y*) should be as small as possible to reduce their effect on the signal image. It well established that random noise can be suppressed by averaging over a time period and the SD of noise σ_n_ is predicted to decline with Δ*T*. However, this is not the case for the background, which is typically caused by adjacent tissue movement due to breathing and heartbeat in small animals. Under these conditions, the variation of SD of background σ_b_ should not be monotonous.

### 2.2. Optimization of window width in temporal averaging

**Figure 1:**
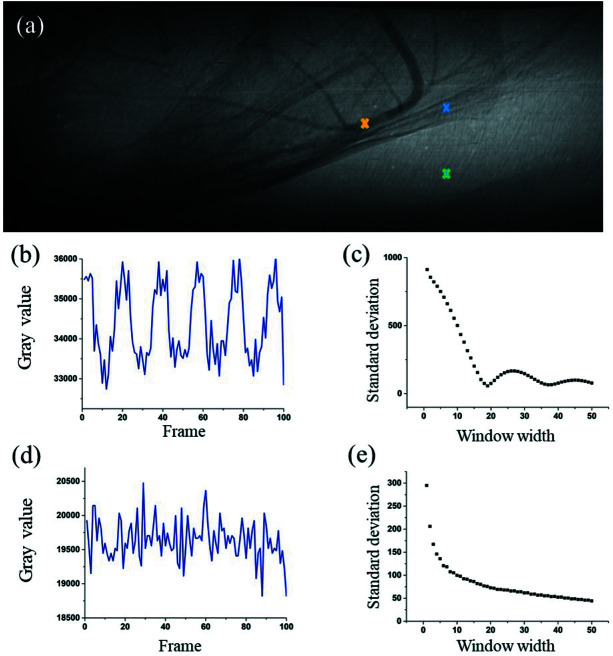
Standard deviation (SD) of signals introduced by tissue motion and background versus window width. (*a*) An original projection in the image sequence collected for angiography of the mouse brain. (*b*) Time-sequenced signal of tissue movements. (*c*) SD of tissue signal versus window width. (*d*) Time-sequenced signal of random noise. (*e*) SD of background noise signal versus window width.

Based on the aforementioned principles, the variations of σ_n_ and σ_b_ with Δ*T* were determined, and the effect of window width on image quality was evaluated quantitatively. Intuitively, the optimized window width should be related to the frequency of tissue movements. Signals introduced by tissue motion and background were investigated, and the results are presented in Fig. 1 ▸. One of the projections in the image sequence is presented in Fig. 1 ▸(*a*), in which the blood vessels, adjacent tissues, and noise are labeled with yellow, blue, and green crosses, respectively. Fig. 1 ▸(*b*) depicts the original signal of tissue movement at the location marked in blue. This periodical signal was due to the breathing and heartbeat of the live mouse. A variety of window widths for temporal averaging were used to process the tissue signals, and the SD was achieved accordingly, as shown in Fig. 1 ▸(*c*). The SD value decreased with an increase in window width until a minimum was reached when the window width value arrived at the fluctuation period of the original signal. Subsequently, the next minimum of SD was reached when the window width increased to another integer multiple of the period of tissue movement.

We further investigated the effect of noise on image quality. Fig. 1 ▸(*d*) presents the temporal distribution of noise at the pixel labeled in green in Fig. 1 ▸(*a*). The SD of background noise versus window width is presented in Fig. 1 ▸(*e*). The SD value decreased monotonically with window width, and the descent rate slowed down after the window width reached 20.

**Figure 2:**
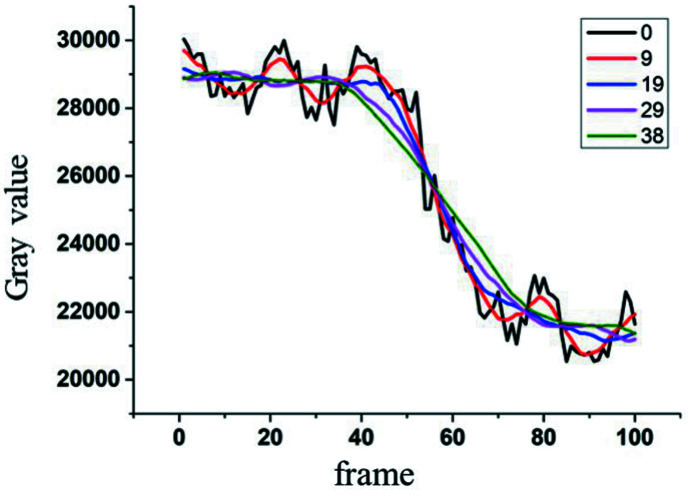
Time-sequenced signals at a vein processed with different window widths.

The fidelity of signals after image processing is essential for evaluating algorithms. The original and processed signals of the blood vessel labeled with a yellow cross in Fig. 1 ▸(*a*) are presented in Fig. 2 ▸. In Fig. 2 ▸, the black curve is the original signal with a periodic fluctuation caused by tissue movement superimposed on a step signal which is due to the migration of contrast agent inside the vessel lumen. The other colored curves represent the results obtained using the temporal averaging method at different window widths. The blue curve corresponding to a window width of 19 exhibited the highest fidelity relative to the trend of the original signal. Further, the fluctuation superimposed on blood vessels, which was due to movement of the adjacent tissues, was also suppressed. This enabled high spatial resolution of microvessels. With an increase in window width, the fluctuation remained suppressed, but the resulting curves were broadened in the time domain, implying a lower fidelity. Accordingly, the value of 19 was identified as the optimal window width for temporal averaging in real-time X-ray imaging of mouse cerebral microvessels *in vivo*.

Based on this analysis, we propose that the best image quality can be obtained when the window width equals the period of tissue movement. At this optimal window width, the fluctuation resulting from tissue movement is reduced to a minimum and the highest fidelity of the processed signal is achieved, while high-frequency noise is suppressed to relatively low levels. In addition, this method is valid for conditions in which the movement of the adjacent tissues is periodic with a period that is substantially shorter than that of the signals and the related amplitude modulated in the signal is no more than the signal intensity. In this regard, the frame rate of the detector should be high enough to depict the full period of movement. For *in vivo* X-ray imaging of the mouse brain, these conditions can be achieved with high success. Accordingly, significant improvements in microvessel images of the mouse brain are expected with the use of this method.

## 3. Experiments and results

Experiments were performed using the BL13W X-ray imaging beamline (Xie *et al.*, 2020 ▸) at Shanghai Synchrotron Radiation Facility. After positioning a double-crystal monochromator downstream of a wiggler source, X-ray energy of 33.2 keV with a flux of 2.38 × 10^10^ photons s^−1^ mm^−2^ was outputted for imaging purposes. The X-ray detector had a pixel size of 6.5 µm (2048 × 788 pixels) and was located 65 cm downstream of the sample. The frame rate of the detector was set to 30 frames s^−1^. For animal preparation, adult male ICR mice weighing 25–30 g were intraperitoneally anesthetized with ketamine (100 mg kg^−1^) and xylazine (10 mg kg^−1^; Sigma, San Louis, Missouri, USA), and an angiographic tube was inserted into the bifurcation of the common carotid artery along the external carotid artery. During data acquisition, 180 µL of non-ionic iodine contrast agent (Ipamiro, Shanghai, China) was injected at a concentration of 280 mgI ml^−1^ into the internal carotid arteries at an injection rate of 133.3 µL s^−1^ under the control of a micro-syringe pump (LSP01-1A; Longerpump, Baoding, China). The injection process of the contrast agent was synchronized with the CCD detector. The detector collected a series of projections as the contrast agent entered the blood vessels, and an image sequence was obtained for the entire perfusion process along the vessels.

**Figure 3:**
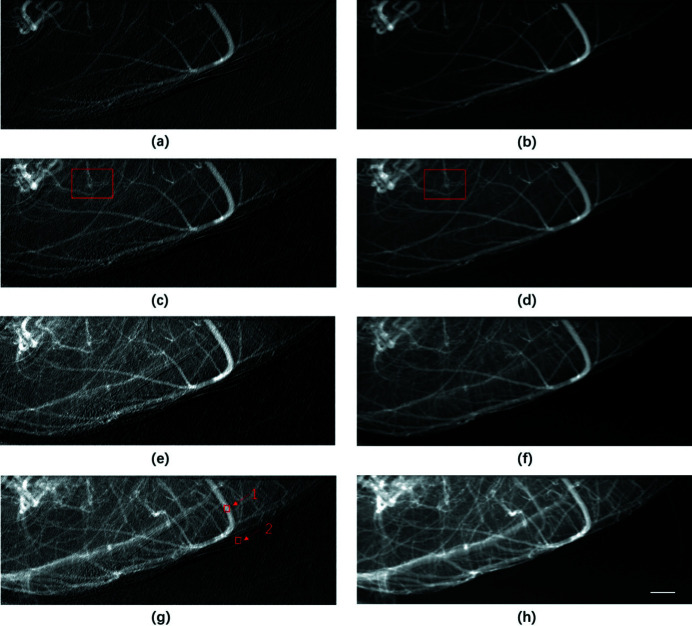
Perfusion process of contrast agent in mouse cerebral blood vessels, where (*a*), (*c*), (*e*), and (*g*) are images of the perfusion process at different phases obtained using the traditional temporal subtraction method, whereas (*b*), (*d*), (*f*), and (*h*) are the counterpart images obtained using PTA. Scale bar: 1 mm.

**Figure 4:**
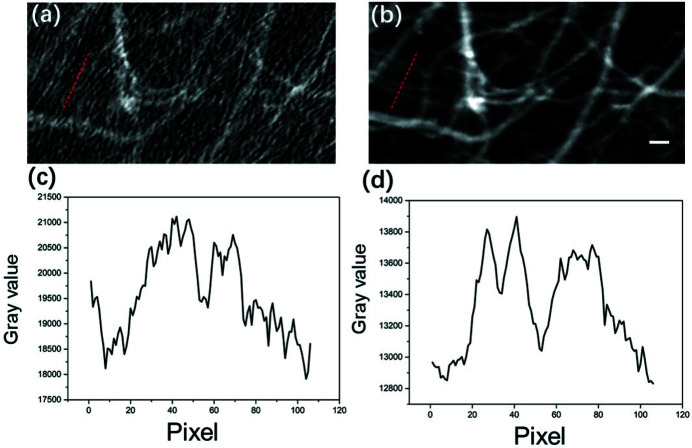
Magnified view of microvessels reconstructed using the temporal subtraction method and PTA, where (*a*) and (*b*) are the areas denoted with a red box in Figs. 3 ▸(*c*) and 3(*d*), respectively. Panels (*c*) and (*d*) are corresponding line profiles at the positions marked by the red dashed lines in (*a*) and (*b*). Scale bar: 200 µm.

The effectiveness of the proposed method for removing motion artifacts was verified using a live mouse. The animal experimental protocols used in this study were approved by the Institutional Animal Care and Use Committee (IACUC) of Shanghai Jiao Tong University, Shanghai, China. Videos S1 and S2 depict the perfusion process of the contrast agent processed using the traditional temporal subtraction method and current temporal averaging method, respectively (see videos in supporting information). As shown in Video S2, the periodic motion of blood vessels observed in Video S1 was successfully eliminated using the proposed method. Detailed analysis can be found in the supporting information. Four typical phases of perfusion were selected to investigate the effects of PTA on the elimination of motion artifacts. The images in Figs. 3 ▸(*a*), 3(*c*), 3(*e*), and 3(*g*) correspond to the perfusion process obtained using the conventional temporal subtraction method, whereas the images in Fig. 3 ▸(*b*), 3(*d*), 3(*f*), and 3(*h*) depict results obtained using PTA. As indicated in Fig. 3 ▸, motion artifacts were clearly eliminated at all phases of the perfusion process, resulting in distinct recovery of signals from microvessels that deteriorated due to tissue movement. Fig. 4 ▸ presents magnified areas denoted with a red box in Figs. 3 ▸(*c*) and 3(*d*). As demonstrated in Figs. 4 ▸(*a*) and 3 ▸(*b*), the CNR of the image was significantly improved by PTA and the microvessels identified using this method were blurred in Fig. 4 ▸(*a*). The profiles of microvessels are provided in Figs. 4 ▸(*c*) and 4(*d*). The evident peaks corresponding to microvessels in Fig. 4 ▸(*d*) were obscured by noise in the profile in Fig. 4 ▸(*c*). According to the full width at half-maximum (FWHM) of the peaks, the diameter of the finest vessel revealed by PTA was approximately 65 µm.

To quantitatively evaluate the image quality of the proposed method, CNR was introduced (Geissler *et al.*, 2007 ▸; Timischl, 2015 ▸). We defined CNR as follows,

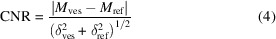

where *M*_ves_ and δ_ves_ are the mean value and SD of the region of interest (ROI) in the blood vessel, respectively, and *M*_ref_ and δ_ref_ are the mean value and SD of the ROI in the background, respectively. As shown in Fig. 3 ▸(*g*), the area indicated by arrows 1 and 2 are the ROIs in the blood vessel and background, respectively. The goal of PTA is to restrict the effects of tissue movement throughout the perfusion process within a short period of time, thereby enabling quasi-dynamic observation of the perfusion. Therefore, CNR of the image frames obtained in a series of periods was determined in order to evaluate the effects of motion suppression. To achieve a stable background for all the frames, the CNR value versus the frames should be as constant as possible. According to equation (4), small and invariable values of *δ*_ves_ and *δ*_ref_ are preferred. In the case of periodical tissue movements, image frames over a whole period are generally required.

**Figure 5:**
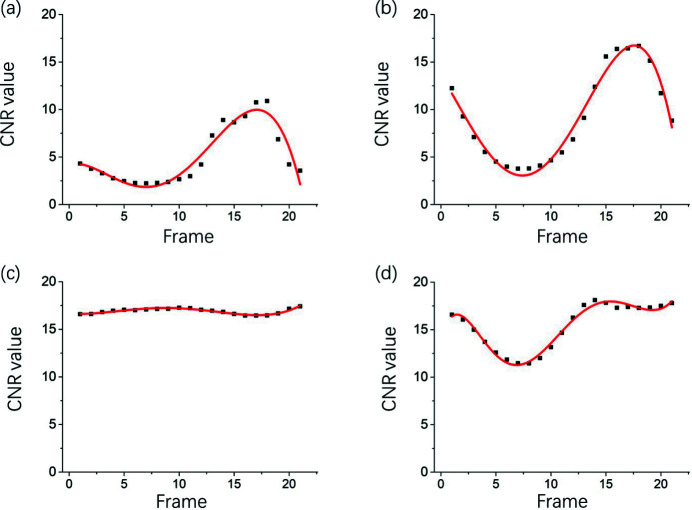
CNR of the retrieved images versus frames obtained continuously after injection of contrast agent, where (*a*) indicates values obtained using the conventional temporal subtraction method and (*b*), (*c*), and (*d*) indicate values obtained using PTA with a window width of 9, 19, and 29, respectively.

Fig. 5 ▸ presents the CNR of the retrieved images versus frames obtained continuously after injection of the contrast agent. As shown in Fig. 5 ▸(*a*), the CNR value of frames obtained using the conventional temporal subtraction method varied with time, implying that background tissue movement was not effectively suppressed, leading to deterioration of the images of microvessels. Figs. 5 ▸(*b*)–5(*d*) present the CNR of image frames processed with a time window width of 9, 19, and 29, respectively. As indicated in Fig. 5 ▸(*b*), CNRs obtained with a width of 9 were larger than those obtained using the conventional method. However, the trends of the curves were similar, suggesting the presence of motion artifacts. Fig. 5 ▸(*c*) depicts the results obtained with a window width of 19, in which the fluctuation in CNR was approximately constant throughout the time period investigated. This suggested that motion artifacts due to background tissues were significantly decreased in this case when the temporal averaging intensity was equal to the period of tissue movement. These findings confirmed the results regarding the optimum window width described in Section 2.2. With an increase in window width to 29, evident fluctuations reappeared, although the CNR increased to a degree, implying an uneven background of the vessel images.

Typically, the time required for PTA reconstruction of 100 frames is approximately 0.25 s in a normal computer with an Inter i9-9900k processor (8 cores, @3.6 GHz). Compared with move contrast angiography, which has an image reconstruction time of approximately 1400 s (Wang *et al.*, 2020 ▸), the proposed method has substantially greater data processing efficiency. This suggests that PTA may enable the quasi real-time observation of microvessels during the perfusion process without the disturbance of motion artifacts.

**Figure 6:**
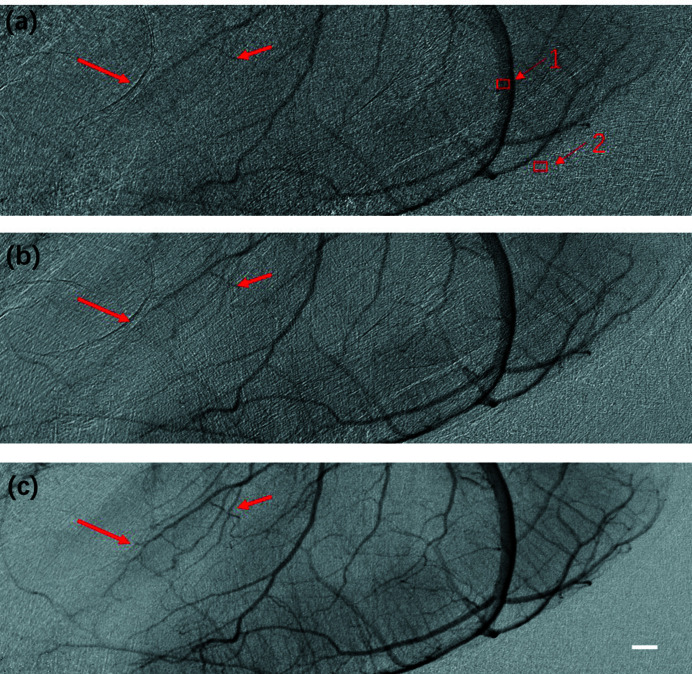
Images of blood vessels with less than a quarter of the photon flux obtained using (*a*) the temporal subtraction method, (*b*) conventional averaging with 10 frames, and (*c*) PTA with a window width of 19. Scale bar: 0.5 mm.

Radiation dose is critical for *in vivo* investigations in live animals. It is well established that a reduction in radiation dose relies on less exposure time or lower photon flux delivered to the animals. Nevertheless, these factors contribute to higher noise levels and a lower CNR. Accordingly, the effect of PTA on vessel imaging under lower photon flux was investigated. As it is challenging to artificially control the total time needed for the contrast agent to pass through the vessels, reducing the exposure time may not be the most effective way to achieve a lower radiation dose. Alternatively, reducing the photon flux incident delivered to the mouse may lead to a direct decrease in radiation dose while exposure time remains the same. During the experiments, an aluminium filter was inserted into the path of the incident X-ray beam to reduce the radiation dose, and the exposure time was maintained at 33 ms. The experimental results revealed that the average gray value of the projection image recorded with an X-ray detector (Hamamatsu, 16-bit dynamic range) decreased from 22000 to appropriately 5000 after the insertion of the filter in the path of the incident X-ray beam, indicating a significant increase in random noise and a reduction in radiation dose. Videos S3 and S4 show the complete perfusion process obtained using the conventional method and PTA, respectively (see videos in supporting information). For convenience, the processed images for a single projection of the perfusion process are presented in Fig. 6 ▸. Fig. 6 ▸(*a*) shows the results obtained using the conventional temporal subtraction method, in which random noise obscured identification of microvessels as indicated by the bold arrows, while the thin arrows denote unaffected vessels. Fig. 6 ▸(*b*) presents the results obtained with conventional averaging of 10 frames. Random noise was effectively reduced, but motion artifacts were still present. Indeed, the microvessels (indicated by the bold arrows) were still faint. After processing using PTA with a window width of 19, the CNR of the processed projection images was substantially improved, and the microvessels (indicated by the bold arrows) could be explicitly distinguished. These results highlight the potential of the PTA method for angiography with a low radiation dose and its applicability in research using animal models of post-stroke drug-induced angiogenesis.

**Figure 7:**
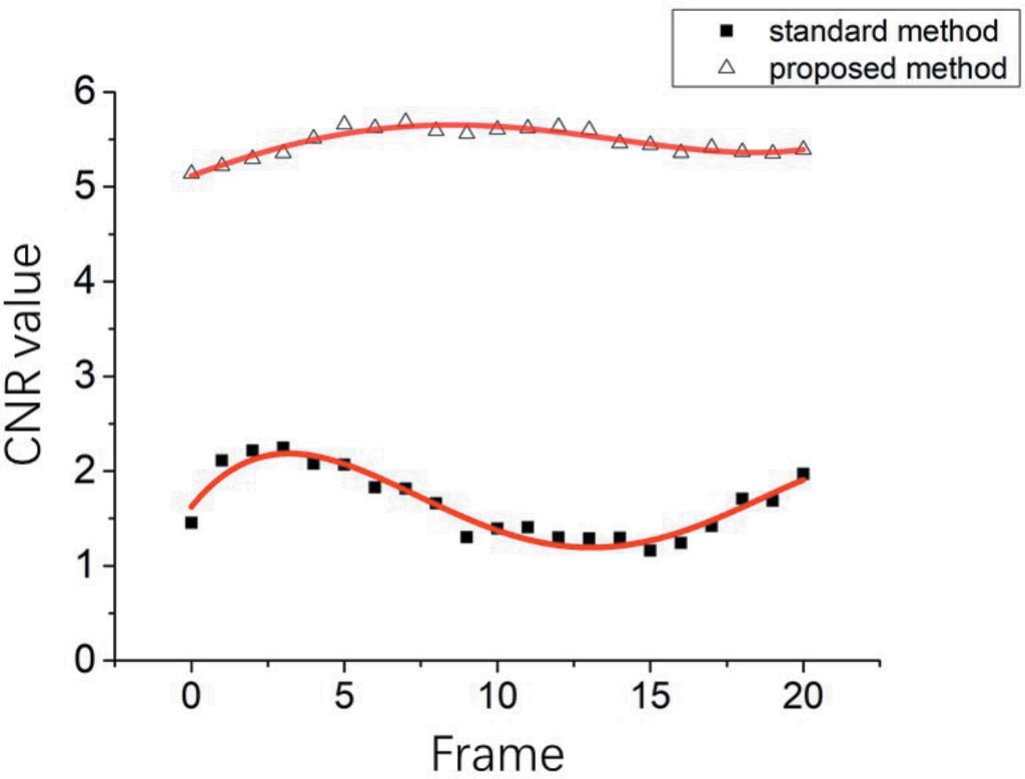
CNRs of the image sequence obtained continuously after the perfusion of contrast agent stabilized. The lower and upper curves correspond to the results obtained using the temporal subtraction method and proposed PTA method, respectively.

CNRs of the sequenced images obtained continuously after the perfusion process was stabilized are plotted in Fig. 7 ▸. The corresponding ROIs of a main blood vessel and background are indicated by Arrows 1 and 2 in Fig. 6 ▸(*a*), respectively. As indicated in Fig. 7 ▸, CNR was significantly improved and fluctuation was suppressed to a constant level by the proposed PTA method compared with that obtained using the traditional temporal subtraction method. The average CNRs of the image frames processed with PTA and the conventional method were 5.47 and 1.65, respectively, indicating a threefold improvement achieved by the proposed method. The CNR SDs during the relevant time period for the PTA method and traditional temporal subtraction were 0.146 and 0.34, respectively, suggesting that a relatively constant image quality could be achieved for all image frames using the proposed method. Collectively, these findings indicate that the PTA method is a promising approach for the *in vivo* imaging of microvessels, with a significant decrease in photon flux incident delivered to the specimen, thereby reducing the radiation dose.

**Figure 8:**
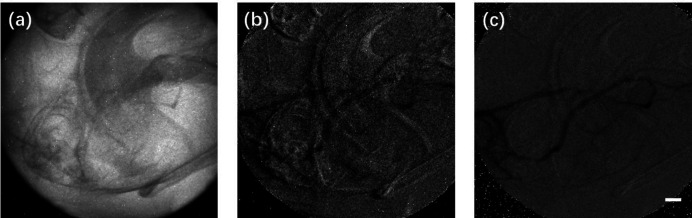
Experimental results obtained using an X-ray tube. (*a*) The primary projection of the cerebral microvessels. (*b*) Image obtained using the conventional temporal subtraction method. (*c*) Image obtained using the PTA method. Scale bar: 1 mm.

X-ray tube-based experiments were also conducted to validate the advantages of the PTA method for angiography with a laboratory X-ray source. A micro-focus X-ray tube with tungsten target (Hamamastu, L8121-03, 75 W) at 80 kV and 500 µA was utilized as an X-ray source. The spot size and divergence of the X-ray source were 50 µm and 43°, respectively. The X-ray detector was the same as that used in experiments with synchrotron X-rays but operated at four binning modes to improve the sensitivity, with an effective pixel size of 26 µm. The detector was placed 10 cm downstream of the sample and set to 5 frames s^−1^ to ensure an exposure time of 200 ms per frame in order to compensate for the low flux density of the X-ray tube during acquisition of the time-sequenced images. As shown in Video S5 in the supporting information, due to the long exposure time and low frame rate, images of microvessels deteriorated notably due to tissue movements. The results are presented in Fig. 8 ▸. Fig. 8 ▸(*a*) depicts the primary projection of the mouse brain. Note that it is difficult to distinguish blood vessels. After processing using the conventional temporal subtraction method, blood vessels remained obscured by considerable motion artifacts. PTA was employed to reconstruct the vessel images. As shown in Fig. 8 ▸(*c*), motion artifacts and random noise were evidently suppressed, and the vessels could be more clearly observed. Video S6 shows the complete perfusion process obtained using PTA. Due to the long exposure time and low spatial resolution, the vessel images were blurred to a degree compared with the results obtained using synchrotron X-rays. These results demonstrate that PTA may be applicable to investigations using a laboratory X-ray source.

## 4. Conclusion

Microvascular dysfunction is associated with various diseases, including cerebral ischemia, hemorrhagic stroke, and hypertension. Rodents are used extensively as animal models for the preclinical investigation of microvascular-related diseases. Motion artifacts prevent currently available methods from achieving real-time observation of microvessels *in vivo*. Here, we developed a dedicated temporal averaging method for real-time imaging of microvessels in live animals by efficiently suppressing the effects of tissue movement and random noise.

In principle, the intensity evolution of vessel images can be divided into three parts: the signal of interest resulting from perfusion of the contrast agent, background fluctuation due to tissue movements, and random noise produced by insufficient photon flux and intrinsic noise from the detector. Efficiently reducing the effects of tissue movement and random noise while maintaining high signal fidelity constitute the objectives of PTA. Temporal subtraction is a traditional method that is typically used to remove perturbations due to a complex background, but elimination of random noise remains a challenge. Temporal averaging is employed to reduce random noise but carries the risk of deteriorating the signals. Accordingly, a dedicated temporal averaging method was developed for the real-time observation of cerebral microvessels in live animals. The window width used for temporal averaging is critical for the implementation of PTA. As tissues tend to move periodically, there may be an optimal window width with which the intensity fluctuations resulting from tissue movement can be largely reduced to time-invariant signals and the effect of motion artifacts can be effectively eliminated. In general, the optimal window width should approximate the period of tissue movements. The fidelity of the signal of interest after temporal averaging is another key issue for PTA. In addition, the period of tissue movement should be much less than that of contrast agent perfusion. In this regard, cerebral imaging in live mice satisfies these conditions and affords an ideal system for the validation of PTA.

In this study, we validated the PTA method experimentally in live mice and demonstrated that motion artifacts resulting from tissue movement were effectively eliminated by the proposed method. Further, random noise was substantially reduced, and the CNR of vessel images was significantly improved. Moreover, the image processing time for 100 frames of 2048 × 788 pixels was approximately 250 ms with a normal computer. This efficient image processing highlights the applicability of PTA for real-time observation of microvessels *in vivo* without the interference of motion artifacts and random noise. Radiation dose is pivotal for the *in vivo* investigation of live animals. Experiments with less than a quarter of the normal photon flux verified that motion artifacts and random noise were effectively suppressed. As a result, cerebral microvessels were successfully identified using PTA, whereas the conventional temporal subtraction and averaging methods were ineffective. This implies that PTA holds the potential for angiography with low radiation dose. Experiments conducted with an X-ray tube verified that the PTA method also applies to *in vivo* investigations in animal models of vessel-related diseases with a laboratory X-ray source.

In conclusion, we developed a PTA method that successfully eliminated the effects of tissue movement and random noise in X-ray imaging of mouse cerebral microvessels *in vivo*. Our proposed PTA method may facilitate the real-time investigation of cerebral microvascular-related diseases using small animal models. Although PTA may be effective for the *in vivo* imaging of cerebral microvessels, it may not be ideal for imaging vessels in the lungs or heart due to the larger amplitude and period of tissue movements in these organs than in the brain. In this regard, the PTA method applies to microvessel imaging in tissues with less movements.

## Supplementary Material

Video S1. DOI: 10.1107/S1600577521012522/mo5250sup1.avi

Video S2. DOI: 10.1107/S1600577521012522/mo5250sup2.avi

Video S3. DOI: 10.1107/S1600577521012522/mo5250sup3.avi

Video S4. DOI: 10.1107/S1600577521012522/mo5250sup4.avi

Video S5. DOI: 10.1107/S1600577521012522/mo5250sup5.avi

Video S6. DOI: 10.1107/S1600577521012522/mo5250sup6.avi

Detailed explanation about the blood vessels no longer move after processed by PTA; Fig. S1. DOI: 10.1107/S1600577521012522/mo5250sup7.pdf

## References

[bb1] Badea, C. T., Drangova, M., Holdsworth, D. W. & Johnson, G. A. (2008). *Phys. Med. Biol.* **53**, R319–R350. 10.1088/0031-9155/53/19/R01 PMC2663796 18758005

[bb2] Balbi, M., Ghosh, M., Longden, T. A., Jativa Vega, M., Gesierich, B., Hellal, F., Lourbopoulos, A., Nelson, M. T. & Plesnila, N. (2015). *J. Cereb. Blood Flow Metab.* **35**, 1445–1453. 10.1038/jcbfm.2015.107 PMC4640303 26058694

[bb3] Candes, E. J., Romberg, J. & Tao, T. (2006). *IEEE Trans. Inf. Theory*, **52**, 489–509.

[bb4] Chen, C., Lin, X., Wang, J., Tang, G., Mu, Z., Chen, X., Xu, J., Wang, Y., Zhang, Z. & Yang, G. Y. (2014). *Stem Cells*, **32**, 2679–2689. 10.1002/stem.1754 24888319

[bb5] Clark, D. P. & Badea, C. T. (2021). *Phys. Med.* **88**, 175–192. 10.1016/j.ejmp.2021.07.005 PMC8447222 34284331

[bb6] Elleaume, H., Charvet, A. M., Corde, S., Est ve, F. & Bas, J. F. L. (2002). *Phys. Med. Biol.* **47**, 3369–3385. 10.1088/0031-9155/47/18/307 12375826

[bb7] Geissler, A., Gartus, A., Foki, T., Tahamtan, A. R., Beisteiner, R. & Barth, M. (2007). *J. Magn. Reson. Imaging*, **25**, 1263–1270. 10.1002/jmri.20935 17520733

[bb8] Hong, G., Lee, J. C., Robinson, J. T., Raaz, U., Xie, L., Huang, N. F., Cooke, J. P. & Dai, H. (2012). *Nat. Med.* **18**, 1841–1846. 10.1038/nm.2995 PMC3595196 23160236

[bb9] Hughes, J. M. & Bund, S. J. (2002). *Exp. Physiol.* **87**, 527–534. 10.1113/eph8702399 12481926

[bb10] Kelly, M. E., Schültke, E., Fiedler, S., Nemoz, C., Guzman, R., Corde, S., Esteve, F., LeDuc, G., Juurlink, B. H. & Meguro, K. (2007). *Phys. Med. Biol.* **52**, 1001–1012. 10.1088/0031-9155/52/4/009 17264366

[bb11] Kidoguchi, K., Tamaki, M., Mizobe, T., Koyama, J., Kondoh, T., Kohmura, E., Sakurai, T., Yokono, K. & Umetani, K. (2006). *Stroke*, **37**, 1856–1861. 10.1161/01.STR.0000226904.96059.a6 16741182

[bb12] Lin, X., Miao, P., Mu, Z., Jiang, Z., Lu, Y., Guan, Y., Chen, X., Xiao, T., Wang, Y. & Yang, G. Y. (2015). *Phys. Med. Biol.* **60**, 1655–1665. 10.1088/0031-9155/60/4/1655 25632958

[bb28] Lin, X., Miao, P., Wang, J., Yuan, F., Guan, Y., Tang, Y., He, X., Wang, Y. & Yang, G.-Y. (2013). *PLoS One*, **8**, e75561. 10.1371/journal.pone.0075561 PMC3782513 24086572

[bb13] Liu, P., Sun, J., Zhao, J., Liu, X., Gu, X., Li, J., Xiao, T. & Xu, L. X. (2010). *J. Synchrotron Rad.* **17**, 517–521. 10.1107/S0909049510018832 20567084

[bb14] Lu, H., Wang, Y., He, X., Yuan, F., Lin, X., Xie, B., Tang, G., Huang, J., Tang, Y., Jin, K., Chen, S. & Yang, G. Y. (2012). *Stroke*, **43**, 838–843. 10.1161/STROKEAHA.111.635235 22223243

[bb15] Maintz, J. B. A. & Viergever, M. A. (1998). *Med. Image Anal.* **2**, 1–36. 10.1016/s1361-8415(01)80026-8 10638851

[bb16] Meijering, E. H. W., Zuiderveld, K. J. & Viergever, M. A. (1999). *Int. J. Comput. Vis.* **31**, 227–246.

[bb17] Perona, P. & Malik, J. (1990). *IEEE Trans. Pattern Anal. Mach. Intell.* **12**, 629–639.

[bb18] Suhonen, H., Porra, L., Bayat, S., Sovijärvi, A. R. & Suortti, P. (2008). *Phys. Med. Biol.* **53**, 775–791. 10.1088/0031-9155/53/3/016 18199914

[bb19] Tan, L. & Jiang, J. (2019). Editors. *Digital Signal Processing*, 3rd ed., pp. 649–726. Academic Press.

[bb20] Timischl, F. (2015). *Scanning*, **37**, 54–62. 10.1002/sca.21179 25533747

[bb21] Wang, F., Zhou, P., Li, K., Mamtilahun, M., Tang, Y., Du, G., Deng, B., Xie, H., Yang, G. & Xiao, T. (2020). *IUCrJ*, **7**, 793–802. 10.1107/S2052252520008234 PMC7467167 32939271

[bb22] Wang, L., Mu, Z., Lin, X., Geng, J., Xiao, T. Q., Zhang, Z., Wang, Y., Guan, Y. & Yang, G. Y. (2017). *Front. Aging Neurosci.* **9**, 359. 10.3389/fnagi.2017.00359 PMC5673661 29163140

[bb23] Wang, L., Zhou, P., Mu, Z., Lin, X., Jiang, L., Cheng, Z., Luo, L., Xu, Z., Geng, J., Wang, Y., Zhang, Z. & Yang, G. Y. (2019). *Transl. Stroke Res.* **10**, 695–704. 10.1007/s12975-019-0687-6 30680639

[bb24] Wilson, D. L., Jabri, K. N. & Aufrichtig, R. (1999). *IEEE Trans. Med. Imaging*, **18**, 22–31. 10.1109/42.750250 10193694

[bb25] Xie, H.-L., Deng, B., Du, G.-H., Fu, Y.-N., Guo, H., Xue, Y.-L., Peng, G.-Y., Tao, F., Zhang, L. & Xiao, T.-Q. (2020). *Nucl. Sci. Tech.* **31**, 102.

[bb26] Yamamoto, M., Okura, Y., Ishihara, M., Kagemoto, M., Harada, K. & Ishida, T. (2009). *J. Digit. Imaging*, **22**, 319–325. 10.1007/s10278-008-9108-1 PMC3043696 18351421

[bb27] Zhang, H., Tan, H., Mao, W.-J., Zhou, J., Fu, Z.-Q., Hu, Y., Xiao, J., Lin, Q.-Y., Shi, H.-C. & Cheng, D.-F. (2019). *Nucl. Sci. Tech.* **30**, 83.

[bb29] Zhou, P. T., Wang, L. P., Qu, M. J., Shen, H., Zheng, H. R., Deng, L. D., Ma, Y. Y., Wang, Y. Y., Wang, Y. T., Tang, Y. H., Tian, H. L., Zhang, Z. J. & Yang, G. Y. (2019). *CNS Neurosci. Ther.* **25**, 748–758. 10.1111/cns.13104 PMC6515698 30784219

