# Supplementary material for: Real-time X-ray imaging of mouse cerebral microvessels in vivo using a pixel temporal averaging method
Source: J Synchrotron Radiat. 2022 Jan 1;29(Pt 1):239–46. doi: 10.1107/S1600577521012522 (PMC8733992; doi:10.1107/S1600577521012522)
Supplement: Supplementary file 7 [file s-29-00239-sup7.pdf]

JOURNAL OF SYNCHROTRON RADIATION

Volume 29 (2022)

Supporting information for article:

Real-time X-ray imaging of mouse cerebral microvessels *in vivo* using a pixel temporal averaging method

Fucheng Yu, Feixiang Wang, Li Ke, Guohao Du, Biao Deng, Honglan Xie, Guoyuan Yang and Tiqiao Xiao

S1. Detailed explanation about the blood vessels no longer move after processed by PTA

In our opinion, the observed apparent movement of blood vessels in Video S1 is contributed by two factors including the movement of blood vessels and complex background. After careful analysis, we came to know that the movement of blood vessels is only a few pixels. The apparent relative movement of blood vessels come from flash of the background (suddenly emerge and disappear). In addition, direction of movement for the blood vessels and the tissue background happens to be opposite. As a result, the observed amplitude of the relative movement between blood vessels and tissue background is enlarged, even though the movement of blood vessels itself is limited. Pixelwise correction cannot stop the movement of blood vessels indeed, but the background is eliminated after proposed method. Accordingly, the contribution of relative movement between blood vessels and background is removed in Video S2.

As to the movement of blood vessels itself, it is removed in Video S2 by the edge averaging. As mentioned above the movement amplitude of the vessels is small compared to the diameter of it. The effect of averaging suppresses the movement and leads to a blurred edge of the vessels especially for the vessels perpendicular to the moving direction. For further analysis, two pixels in the movie are selected to explain the effect of temporal averaging on the vessel movement. These two pixels represent the middle and boundary of the blood vessel. Figure S1(a) is a time-sequenced signal at the center of blood vessel, it mainly consists of three kinds of signals, i.e. periodic fluctuation caused by tissue movement, step-like signal of agent perfusion and random noises. After processed by PTA, this signal is transformed into a step-like signal shown in figure S1(b), which means tissue movement and random noises are all suppressed. Figure S1(c) shows the time sequenced signal at the boundary of blood vessels, which is mainly contributed by tissue movement and random noises. After the 50th frame, periodic fluctuations with large amplitude emerged, which is actually resulted by the jitter of blood vessels perfused with contrast agent. As shown in figure S1(d), the periodical signal is transformed into a step-like signal similar to that shown

in figure S1(b). This implies that grayscale fluctuation which refers to movement of vessels is suppressed also at the boundary. As a result, the vessels look like still.

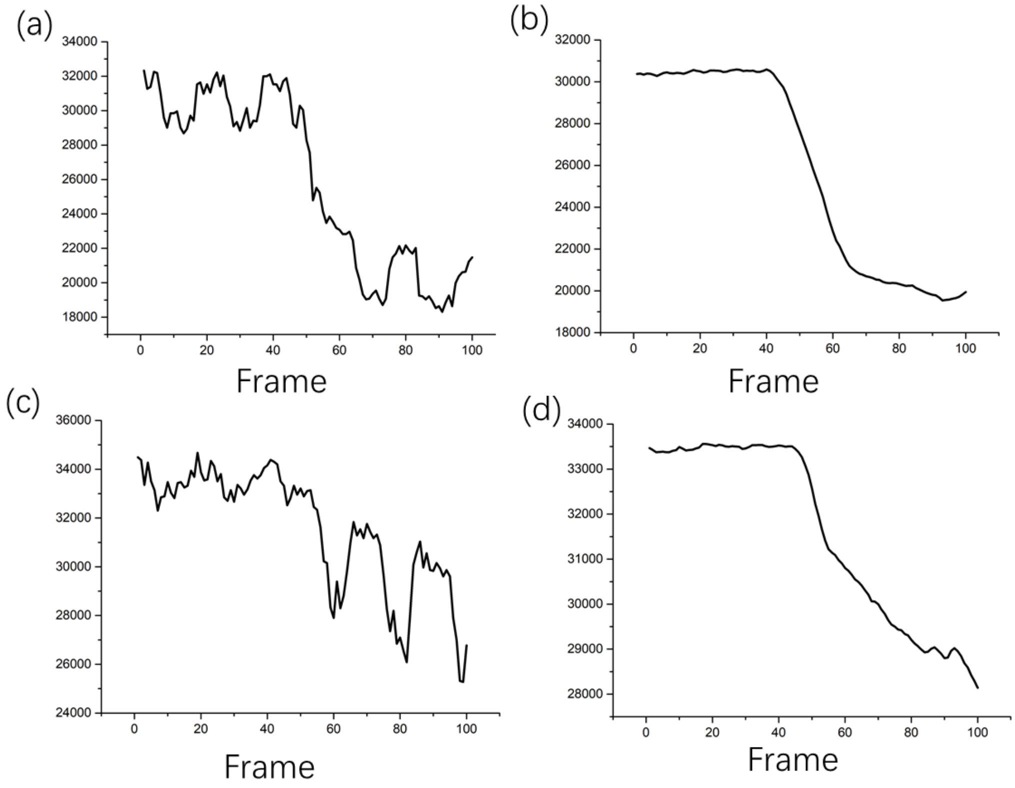

Figure S1 Figure S1. Time-sequenced signals at a blood vessel, where (a) signal at the center of blood vessel, (b) the signal (a) processed by PTA, (c) signal at the boundary of blood vessel, (d) the signal (c) processed by PTA.
